# Effects of laser processing parameters and scanning angle on damage morphology and residual tensile properties of CFRP laminates with a continuum damage mechanics model

**DOI:** 10.1371/journal.pone.0361461

**Published:** 2026-11-06

**Authors:** Wei Liang, Mingwei Ren, Yunbo Chen, Lingli Zuo, Peng Pan

**Affiliations:** 1 School of Mechanical Engineering, University of Science and Technology Beijing, Beijing, China; 2 China Academy of Machinery Science and Technology Group Co., Ltd, Beijing, China; 3 Beijing National Innovation Institute of Lightweight Ltd, Beijing, China; King Mongkut's University of Technology North Bangkok, THAILAND

## Abstract

Laser processing of carbon fiber-reinforced polymer (CFRP) composites offers significant advantages in precision and efficiency, yet the thermal damage it induces remains a critical concern for structural integrity. This study investigates the effects of laser drilling and cutting on the damage morphology and residual tensile properties of T300-3K/epoxy CFRP laminates, with systematic variation of laser power, irradiation duration, scanning angle, and scanning distance across 21 processing conditions. White-light interferometric microscopy characterized three-dimensional ablation topography, and uniaxial tensile testing quantified mechanical degradation. For drilled specimens, increasing power from 250 W to 1000 W enlarged the ablation crater diameter by approximately 200% (1007–3021 μm), while extending irradiation duration from 0.5 s to 1.0 s produced a further 37% increase. For cut specimens, perpendicular scanning induced a broader heat-affected zone and more severe interfacial disruption than fiber-parallel scanning due to anisotropic thermal conductivity, with the maximum surface height difference reaching 4259 μm at 500 W and 90°. Residual tensile strength decreased monotonically with increasing energy input: drilled specimen strength declined from 735 MPa to 293 MPa (a reduction of over 60% to the pristine strength of 954 MPa); cut specimen strength at 500 W decreased from 466 MPa at 0° to 233 MPa at 90° due to progressive fiber severance, with cut specimens consistently weaker than drilled counterparts owing to kerf-induced stress concentration. A CDM-based empirical model integrating all four parameters was developed; nonlinear regression yielded A = 0.0266187, m = 0.464431, n = 0.645373, α = 0.0745099, β = 0.22664, and R^2^ = 0.9151, providing a validated quantitative tool for structural integrity assessment of laser-processed CFRP components.

## 1 Introduction

Carbon fiber reinforced polymers (CFRPs) are widely adopted in aerospace primary and secondary load-bearing structures owing to their high specific strength, high specific stiffness, and excellent fatigue resistance [1–5]. In manufacturing and maintenance scenarios, laser processing—including drilling and cutting—has emerged as a preferred alternative to conventional mechanical machining due to its non-contact nature, absence of tool wear, and high geometric precision [6–10]. However, the inherently low thermal decomposition temperature of the epoxy matrix makes CFRP composites highly susceptible to laser-induced thermal damage, manifesting as matrix pyrolysis, interfacial debonding, carbon fiber oxidation, and delamination [11–14]. These thermally induced defects directly compromise the structural integrity and residual load-bearing capacity of processed components.

The extent of laser-induced damage in CFRP is governed by laser parameters including power, irradiation duration, and energy density. Increasing laser power or exposure time enlarges the heat-affected zone (HAZ), accelerates ablation, and degrades residual mechanical properties [15–19]. Continuous-wave (CW) lasers, with their sustained high average power output, generate more severe and spatially extensive thermal damage compared with pulsed systems, making parameter optimization critical for structural applications [15–17]. Despite growing research on laser ablation morphology and macroscopic strength degradation, the quantitative relationships between processing parameters, three-dimensional damage topography, and residual tensile performance remain insufficiently established, particularly under combined drilling and cutting conditions.

A further complexity arises from the anisotropic thermal conductivity of woven CFRP laminates. Heat preferentially conducts along carbon fibers, so the scanning angle relative to fiber orientation directly determines the spatial extent of thermal damage and the number of load-bearing fibers severed during cutting [18–20]. The anisotropic thermal response of CFRP to laser irradiation as a function of scanning angle has been examined in a number of studies, though the scope of existing work is predominantly limited to thermal and morphological characterization, with systematic mechanical degradation data remaining scarce. Wang et al. investigated the heat transfer and ablation mechanism of CFRP under nanosecond pulsed laser processing across five scanning directions (0°, 30°, 45°, 60°, and 90°) relative to the fiber axis, establishing that laser scanning parallel to the fiber direction produces deeper and narrower ablation grooves owing to the preferential heat conduction along the fiber axis, while transverse scanning generates broader and shallower damage zones [20]. However, this study was conducted at fixed laser energy and scanning speed, and no residual mechanical property data were reported, leaving the relationship between scanning angle and tensile strength degradation unquantified. An et al. examined the coupled effects of scanning angle and scanning speed on the surface morphology, physicochemical properties, and mechanical performance of nanosecond laser-treated CFRP intended for adhesive bonding pretreatment, finding that a 45° scanning direction at 3000 mm/s optimally removes surface resin while avoiding carbon fiber damage [3]. Nevertheless, this work focused on surface preparation quality rather than the structural load-bearing capacity of the laminate, and the angle range was not systematically varied against laser power to yield a cross-parametric degradation map. Xu et al. developed a numerical model accounting for the heterogeneity and anisotropy of CFRP to analyze the laser material removal mechanism under five scanning directions (0°, 30°, 45°, 60°, 90°), demonstrating that anisotropic heat transfer leads to distinctly different ablation morphologies depending on fiber orientation [21]. While this study provided valuable mechanistic insight, it was confined to ablation geometry prediction and did not extend to residual tensile property assessment. In the context of laser cutting for structural applications, Keuntje et al. developed a three-dimensional finite element model to predict the spatial and temporal temperature fields during laser cutting of unidirectional CFRP at cutting angles of 45° and 90° relative to the main fiber orientation, confirming that fiber orientation measurably influences the temperature distribution and HAZ extent [22]; however, only two discrete angles were examined, mechanical testing was not performed, and the study did not address woven laminates or the combined influence of power and angle on residual strength. Most recently, Wenfeng et al. explored scanning directions of 0°, 30°, 45°, 60°, and 90° in the context of nanosecond laser recycling of CFRP, demonstrating that heat conduction pathways critically govern thermal damage extent and regenerated fiber quality, yet the objective was fiber recovery rather than structural integrity assessment of the processed laminate [23]. Existing studies have predominantly examined fiber-parallel or fiber-perpendicular scanning in isolation, and no unified quantitative framework has been established that relates scanning angle to residual mechanical properties across a continuous range of processing conditions—a gap that limits the predictive capability available to engineers designing laser processing protocols for load-bearing CFRP structures.

Continuum damage mechanics (CDM) provides a physically grounded framework for quantifying material degradation through a scalar damage variable D, and has been successfully applied to impact and fatigue damage in composites. The MLT model introduced by Matzenmiller, Lubliner, and Taylor employs mode-specific damage variables for fiber fracture, matrix cracking, and shear failure to drive progressive stiffness reduction, establishing the foundational constitutive framework for anisotropic damage in fiber-reinforced composites [24]. At the ply level, Ladevèze and Le Dantec formulated separate damage variables for transverse matrix microcracking and fiber–matrix debonding, with stiffness reductions derived from thermodynamic potentials and identified through systematic laminate tests [25]. These frameworks have since been extended to impact loading—where CDM coupled with cohesive zone models accurately predicts both damage extent and residual compressive strength after low-velocity impact [26]—and to thermally influenced scenarios, in which temperature-dependent CDM formulations have successfully reproduced the progressive failure of CFRP under simultaneous thermal and mechanical loading across a range of impact energies [27]. Most relevantly to the present context, CDM has recently been adapted to lightning-strike-induced coupled thermal–mechanical damage in CFRP, quantifying the synergistic stiffness degradation arising from thermal ablation and thermally driven expansion [12]. Taken together, these applications confirm CDM as a mature framework for progressive degradation prediction across diverse damage-inducing mechanisms in CFRP laminates.

In this study, the effects of laser power, irradiation duration, and scanning angle on the ablation morphology and residual tensile strength of T300-3K/epoxy CFRP laminates were systematically investigated under both drilling and cutting modes. White-light interferometric microscopy was employed to characterize three-dimensional damage topography, and uniaxial tensile testing was used to quantify mechanical degradation. Existing models are generally limited to a single laser-processing operation, such as drilling or cutting. In contrast, the key novelty of this study lies in developing an empirical model based on CDM that provides a unified framework for predicting damage under both laser-drilling and laser-cutting conditions. The proposed model therefore offers a practical tool for assessing the structural integrity of laser-processed CFRP components.

## 2 Materials and methods

### 2.1 CFRP composite

The CFRP material used in this study comprised T300-3K carbon fibers (fiber diameter: 8 μm) as the reinforcement and epoxy resin as the matrix, with a fiber volume fraction of 62% and a resin volume fraction of 38%, as shown in Fig 1. Laminates with dimensions of 500 mm × 10 mm × 2 mm were fabricated from ten plies, each 0.2 mm thick, with a stacking sequence of [0°/90°/0°/90°]s, yielding an orthogonal multidirectional fiber architecture. The laminates were manufactured via prepreg lay-up followed by hot-press curing. Key physical properties of the material are summarized in Table 1.

**Table 1:** Physical property specifications of the carbon fiber and epoxy resin used in the CFRP.

Constitute	Density (g/cm^3^)	Content (%)	Conduction (W/(mk))	Gasification temperature (℃)
Carbon fiber	1.76	62	8	3700
Epoxy resin	1.20	38	0.2	500

**Fig 1:**
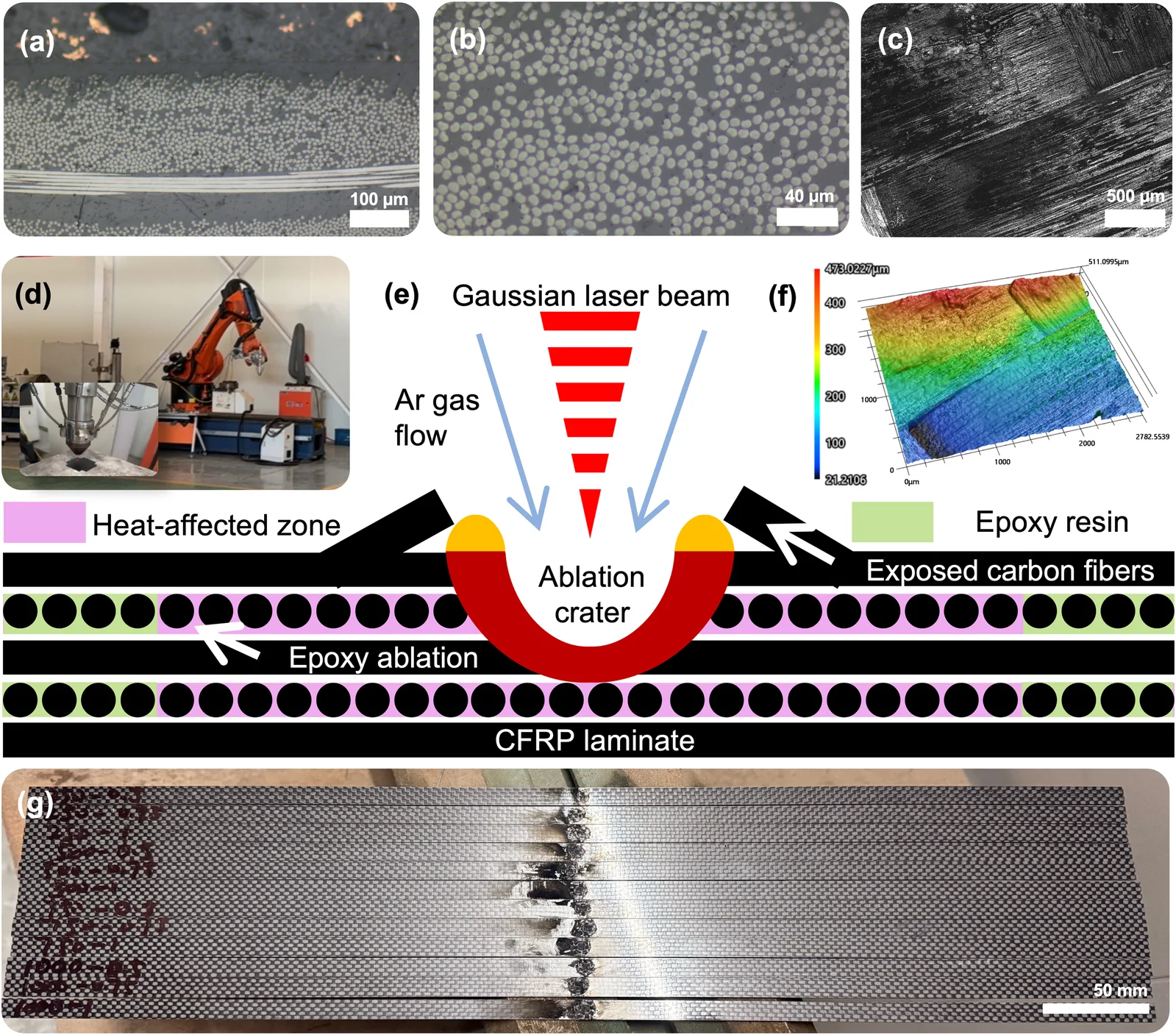
Schematic illustration of the CFRP laser ablation experimental setup. (a) Microstructure of CFRP laminate; (b) morphology of individual carbon fibers; (c) as-received CFRP morphology; (d) laser processing system configuration; (e) laser ablation mechanism; (f) as-received CFRP 3D surface topography;(g) laser-processed CFRP specimens.

### 2.2 Laser processing

A CAM-EHLA5000 ultra-high-speed laser cladding system equipped with an LDF3000−40 multimode continuous-wave laser source was used to perform drilling and cutting operations on CFRP laminates (Fig 1). The system delivered a maximum output power of 3300 W and produced a Gaussian beam with a spot diameter of 3.8 mm. During ablation, the Gaussian beam simultaneously vaporized both the carbon fibers and the epoxy matrix. High-purity nitrogen (≥99%) was supplied as the shielding gas at a flow rate of ~10 L/min throughout all processing operations. Two experimental schemes were designed to investigate the effects of drilling and cutting modes on the residual tensile properties of the laminates.

In the drilling experiments, laser power (P) was varied at 250, 500, 750, and 1000 W, and irradiation duration (t) was set to 0.5, 0.75, and 1.0 s. In the cutting experiments, the laser beam traversed a 10 mm path (scanning length, l = 10 mm) at a constant scanning speed of 10 mm/s; laser power (P) was set to 200, 250, and 500 W, and the angle (θ) between the scanning direction and the tensile loading direction was varied at 0°, 45°, and 90°. This yielded 21 experimental groups in total, as detailed in Table 2 and 3.

**Table 2:** Laser drilling processed CFRP specimens with different parameters.

Parameters		Laser power (W)			
		250	500	750	1000
Scanning time (s)	0.50	Specimen 1−1	Specimen 2−1	Specimen 3−1	Specimen 4−1
	0.75	Specimen 1–2	Specimen 2−2	Specimen 3−2	Specimen 4−2
	1.00	Specimen 1–3	Specimen 2–3	Specimen 3−3	Specimen 4−3

**Table 3:** Laser cutting processed CFRP specimens with different parameters.

Parameters		Laser power (W)		
		200	250	500
Scanning angle (º)	0	Specimen 5−1	Specimen 6−1	Specimen 7−1
	45	Specimen 5−2	Specimen 6−2	Specimen 7−2
	90	Specimen 5−3	Specimen 6−3	Specimen 7−3

### 2.3 Characterizations

After laser drilling and cutting, specimens were cleaned with argon gas to remove residual debris and ablation by-products. Ablation morphology and peak-to-valley surface height were characterized using a Keyence VK-X3000 white-light interferometric laser microscope, and microstructural features were examined by scanning electron microscopy (SEM) using a Phenom Pro instrument.

Tensile properties were evaluated using a WDW-300E universal testing machine (Jinan Shidai Shijin). Specimens had dimensions of 500 mm × 10 mm × 2 mm and were tested at a crosshead speed of 0.1 mm/s under ambient conditions (24 °C, 35% RH). The pristine CFRP specimens yielded an average tensile strength of 954 MPa, with individual values of 973, 987, and 902 MPa. Residual tensile strength of laser-processed specimens was assessed under identical conditions. At least three replicate tests were performed for both the pristine and each laser-treated group to ensure reproducibility.

### 2.4 Continuum damage mechanics model

Accurate prediction of structural integrity loss in laser-processed CFRP laminates requires a unified modeling framework capable of capturing the combined influence of thermal and geometric processing variables. In the present work, this is achieved through an empirical formulation grounded in CDM, a well-established approach in which material degradation is represented by a scalar internal state variable D that evolves from zero in the virgin state to unity at complete failure. Rather than resolving individual microscale failure events—such as fiber breakage, matrix pyrolysis, or interfacial debonding—CDM operates at the continuum level by quantifying the macroscopic consequence of these mechanisms: the progressive reduction in effective load-bearing area and, correspondingly, in measurable mechanical response.

Within this framework, the experimentally accessible residual tensile strength σ serves as the primary observable linked to the damage state. The effective stress principle yields the following constitutive relation between σ, the reference (undamaged) tensile strength σ₀, and the damage variable D:


$$\begin{array}{c} \begin{array}{c} \sigma =\sigma_{\text{0}}\times (\text{1}-D) \end{array} \end{array}$$ (1)

Accordingly, the damage variable D serves as the central predictive quantity in this framework, and its governing parameters are determined through regression against experimental data to enable high-fidelity strength prediction across the full range of processing conditions examined in this study.

## 3 Results and discussion

### 3.1 Microstructural characterization of laser-processed CFRP

Fig 2 presents the macroscopic surface morphologies of laser-drilled CFRP specimens under varying laser powers and irradiation durations. All specimens exhibited circular damage zones with well-defined boundaries. For Specimen 1−1 (250 W, 0.5 s), pronounced carbonization and soot deposition were observed around the drilled hole, particularly near the upper edge of the irradiation zone, attributable to redeposition of gaseous pyrolysis products from epoxy resin decomposition. As laser power and irradiation duration increased, carbonized deposits progressively diminished and hole boundaries became more distinct. Increasing laser power from 250 W to 1000 W induced severe carbon fiber ablation, with the surface crater diameter expanding from 1007 μm (Specimen 1−1) to 3021 μm (Specimen 4−1), an increase of approximately 200%, directly demonstrating the positive correlation between incident laser energy and ablation damage. Extending the irradiation duration similarly intensified damage: increasing exposure from 0.5 s to 1.0 s enlarged the crater diameter from 1007 μm (Specimen 1−1) to 1382 μm (Specimen 1−3), an increase of approximately 37%, accompanied by more severe matrix decomposition and fiber degradation. Under high-power, prolonged irradiation conditions (Specimens 4−2 and 4−3), through-thickness perforation was observed, indicating that elevated laser power combined with extended exposure can induce severe thermal damage, internal delamination, and through-thickness failure. These findings establish a clear relationship among laser power, irradiation duration, and surface damage characteristics, providing a basis for understanding the microstructural response of CFRP under controlled laser irradiation and for subsequent damage evolution modeling. A similar pattern is found in nanosecond laser drilling of CFRP [28]: increasing laser power enhances material removal efficiency, increases exit aperture size, and decreases taper, yet this improvement is accompanied by a widening of the HAZ at both the entrance and exit surfaces.

**Fig 2:**
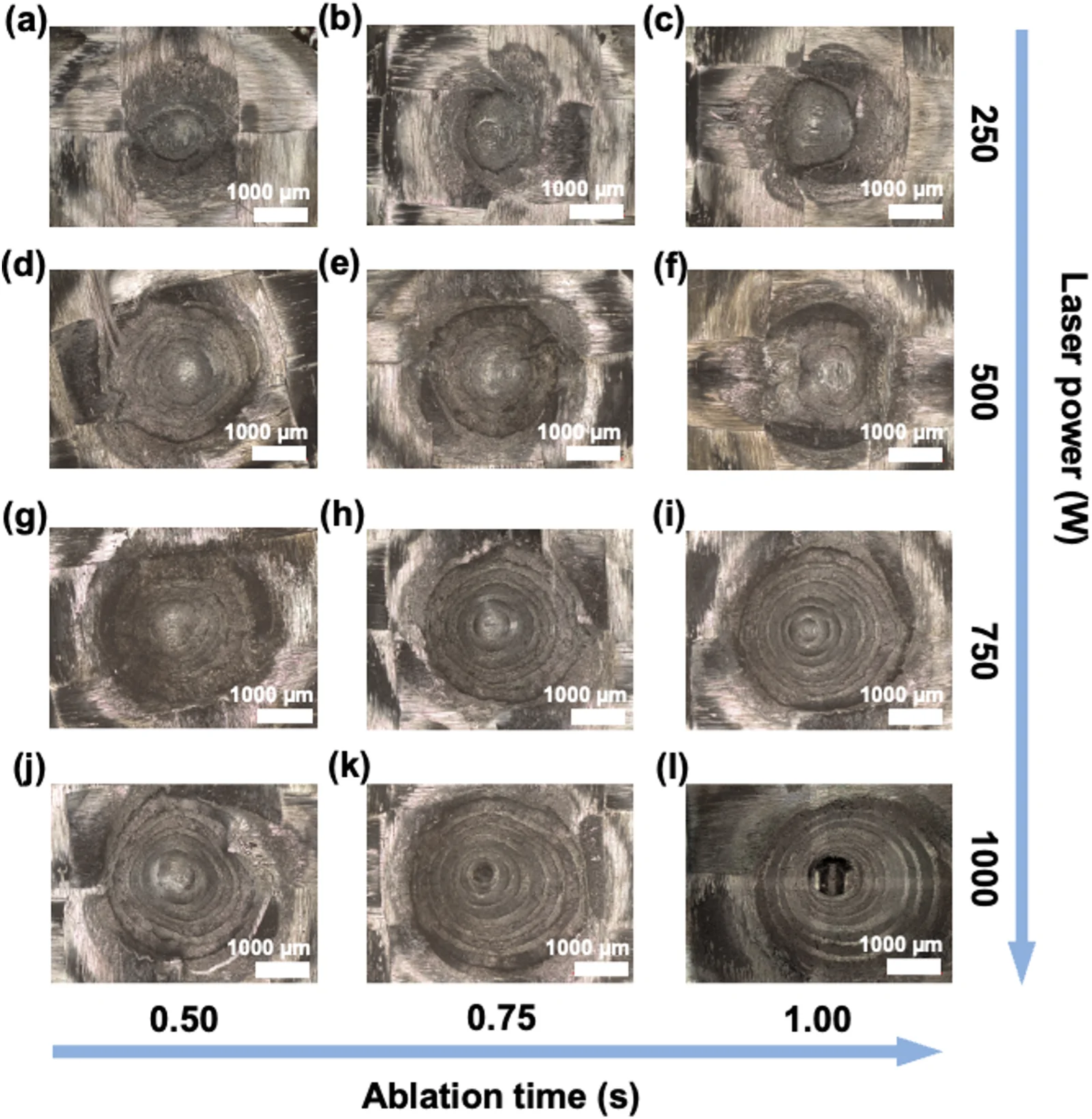
Surface morphologies of laser-drilled CFRP specimens as a function of laser power and ablation duration: (a) 250 W, 0.50 s; (b) 250 W, 0.75 s; (c) 250 W, 1.00 s; (d) 500 W, 0.50 s; (e) 500 W, 0.75 s; (f) 500 W, 1.00 s; (g) 750 W, 0.50 s; (h) 750 W, 0.75 s; (i) 750 W, 1.00 s; (j) 1000 W, 0.50 s; (k) 1000 W, 0.75 s; (l) 1000 W, 1.00 s.

White-light interferometric laser microscopy characterization of the laser-drilled specimens is presented in Fig 3. At 250 W and 0.5 s, a pronounced ablation crater formed on the CFRP surface, yet carbon fiber bundles remained intact. In the height maps, dark blue denotes the lowest regions and dark red the highest. Fractured carbon fibers are concentrated in the dark red regions, where epoxy resin evaporation disrupted the interlaminar bonding structure. Orange-colored regions surrounding the irradiated area indicate localized fiber protrusion caused by resin vaporization. As shown in Fig 3(a), the peak-to-valley height reached 646.297 μm. Comparing Fig 3(e) (Specimen 1–2) and 3(i) (Specimen 1–3) under the same 250 W condition, increasing the irradiation duration from 0.75 s to 1.00 s preserved the dark blue central region but caused significant expansion of the yellow and orange zones, reflecting a larger area and greater depth of resin vaporization. Correspondingly, the peak-to-valley height increased from 696.979 μm to 933.772 μm. Analogous trends were observed at higher laser powers. For specimens exhibiting complete through-thickness perforation (Fig 2(h) and 2(l)), the peak-to-valley heights in Fig 3(h) and 3(l) further increased from 2560.324 μm to 3392.251 μm. Fig 3(m) shows the evolution of the peak‑to‑valley surface height as a function of ablation time and laser power in laser drilling.

**Fig 3:**
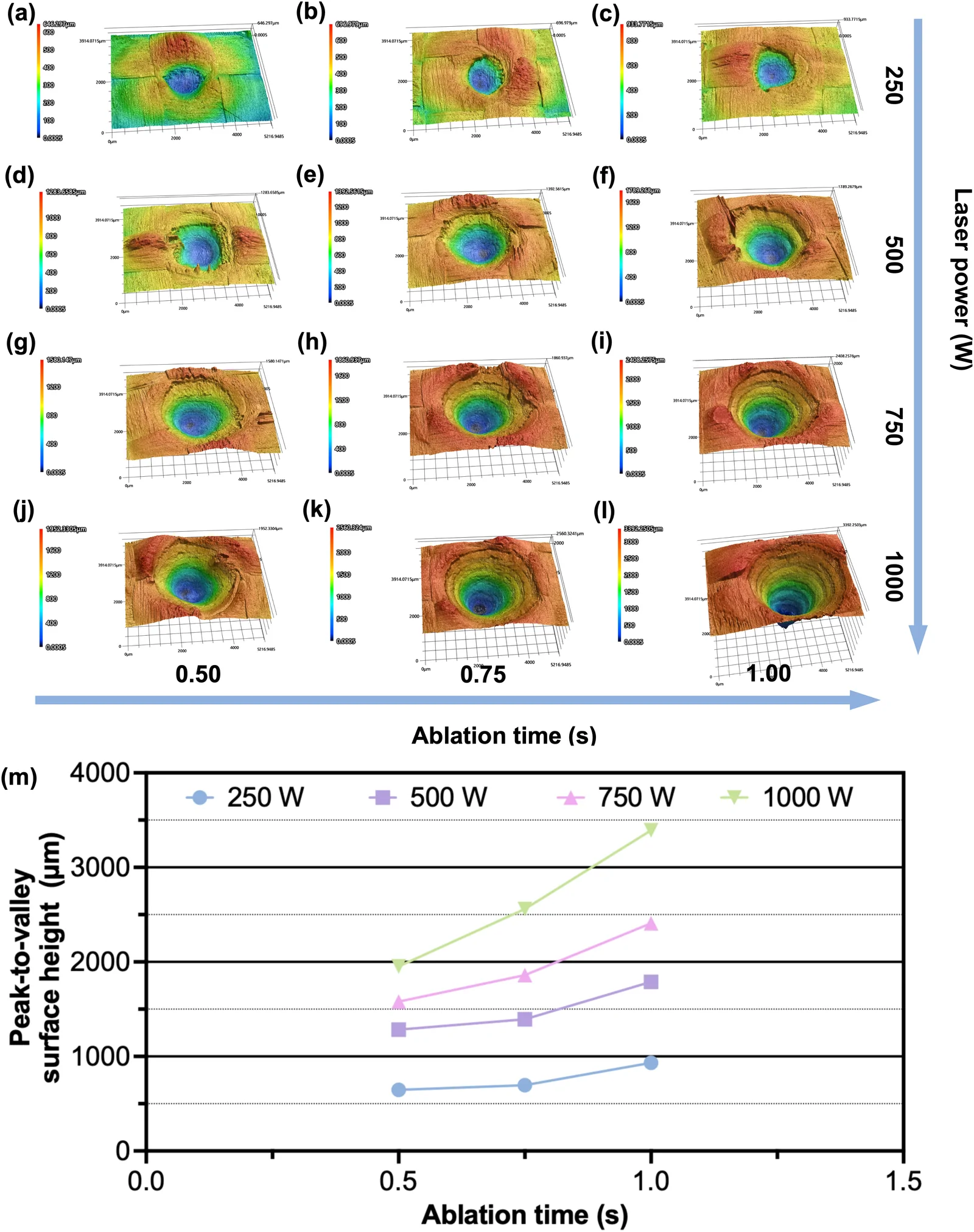
Three-dimensional surface topography of laser-drilled CFRP specimens as a function of laser power and ablation duration: (a) 250 W, 0.50 s; (b) 250 W, 0.75 s; (c) 250 W, 1.00 s; (d) 500 W, 0.50 s; (e) 500 W, 0.75 s; (f) 500 W, 1.00 s; (g) 750 W, 0.50 s; (h) 750 W, 0.75 s; (i) 750 W, 1.00 s; (j) 1000 W, 0.50 s; (k) 1000 W, 0.75 s; (l) 1000 W, 1.00 s; (m) Peak-to-valley surface height trends.

It is evident that both parameters exhibit a positive correlation with the peak‑to‑valley height of the ablation crater. These results indicate that laser-induced thermal damage extended well beyond the laser spot, as the high thermal conductivity of carbon fibers facilitated heat transfer into the surrounding epoxy matrix, driving extensive matrix vaporization and enlarging the heat-affected zone. In ultrashort pulsed laser drilling of CFRP, the “bowl‑bottom effect” is also observed [29]: drilling efficiency deteriorates with increasing depth, and closer to the hole exit, the processing time becomes longer, thermal damage becomes more severe, and the taper becomes larger. Its formation involves three stages. In Stage I, the taper is small, originating from the Gaussian energy distribution of the laser beam. In Stage II, due to shielding by the upper slope surface, the focused spot area and its projected area expand, causing a sharp drop in laser fluence and accumulation of taper. In Stage III, only a small portion of the laser beam reaches the bottom for secondary processing, ultimately forming a large bowl‑shaped taper at the exit.

Fig 4 presents the macroscopic surface morphologies of laser-cut CFRP specimens under varying laser powers and scanning angles. Since both the 0° and 90° cutting directions are perpendicular to the orthogonal woven fiber orientation, the resulting microstructures are highly similar; only the 90° direction is therefore shown. At 200 W (Specimen 5−1, Fig 4(a)), three carbon fiber bundles were severed with an arc-shaped fracture profile, and considerable carbon fiber debris accumulated at the bottom of the ablation groove. Increasing the power to 250 W (Specimen 5−2, Fig 4(b)) transformed the fracture morphology from arc-shaped to serrated, with multiple fiber layers cut within the kerf. At 500 W (Specimen 5−3, Fig 4(c)), the fracture morphology evolved into a relatively flat and uniform profile, and the kerf completely penetrated the full laminate thickness. Overall, increasing laser power progressively yielded smoother and flatter cutting morphologies.

**Fig 4:**
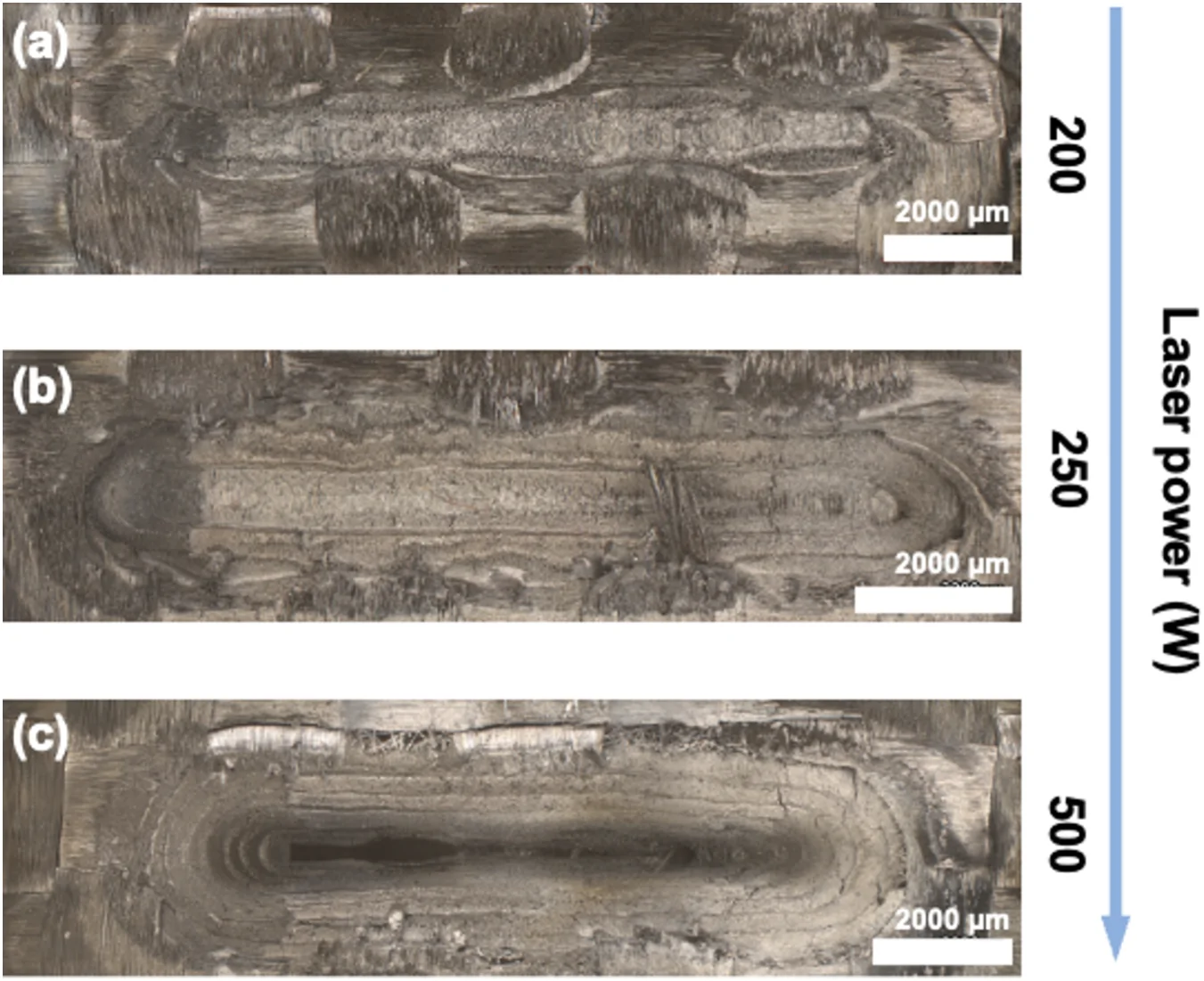
Surface morphologies of laser-cut CFRP specimens at a 90° scanning angle as a function of laser power. (a) 200 W; (b) 250 W; (c) 500 W.

Fig 5 presents the three-dimensional surface morphologies of laser-cut CFRP specimens under varying processing conditions. Distinct groove-like ablation regions are evident in all specimens, accompanied by clear material removal and thermal damage along the laser scanning path. In the height maps, blue and green regions correspond to the deepest ablation zones, while yellow and red regions represent elevated areas associated with molten material accumulation and matrix degradation. As laser energy input increased, the ablation grooves deepened and widened, reflecting enhanced material removal and intensified thermal erosion: the peak-to-valley height difference increased from 588.052 μm at 200 W (Specimen 5−3) to 968.305 μm at 250 W (Specimen 6−3), and further to 4259.334 μm at 500 W (Specimen 7−3). Concurrently, the groove morphology became progressively more continuous and uniform, indicating more complete carbon fiber fracture and epoxy resin vaporization at higher energy levels. Pronounced edge uplift and irregular surface fluctuations around the grooves suggest severe thermal stress concentration and matrix pyrolysis during irradiation. These results further confirm that laser-induced thermal effects are not confined to the direct irradiation zone but propagate along the thermally conductive carbon fibers, enlarging the heat-affected zone and aggravating internal structural damage within the CFRP laminate. Research on thermal damage in nanosecond UV laser cutting of CFRP indicates that the coupling effect of heat conduction and heat accumulation is the primary cause of damage aggravation [30]. Both experimental and simulation results reveal that the thermophysical properties of the resin and fibers differ significantly—the resin vaporizes prior to the fibers, leaving the fibers exposed; meanwhile, heat conduction along the fiber axial direction is far more efficient than in the radial direction, resulting in a substantially greater extension of resin damage along the axial direction.

**Fig 5:**
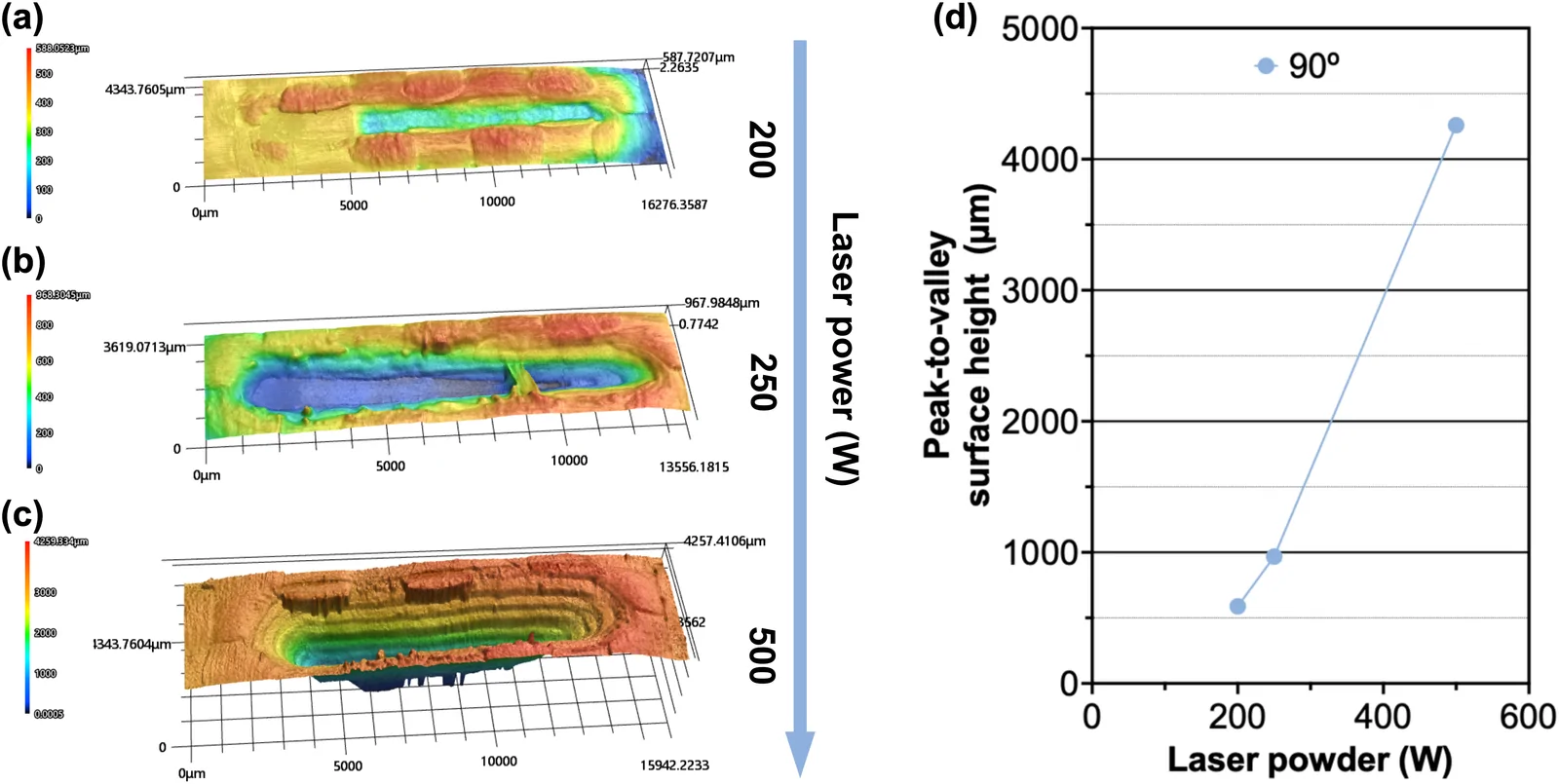
Three-dimensional surface topography of laser-cut CFRP specimens at a 90° scanning angle as a function of laser power. (a) 200 W; (b) 250 W; (c) 500 W; (d) Peak-to-valley surface height trends.

Fig 6 presents the macroscopic surface morphologies of laser-cut CFRP specimens processed at a 45° scanning angle under varying laser power conditions. All specimens exhibited a single ablation groove along the scanning path, yet notable differences in groove depth and thermal damage characteristics were observed. At 200 W (Fig 6(a)), a shallow ablation groove formed alongside visible detachment of the woven carbon fiber architecture in the upper-left region, accompanied by substantial fiber fracture. At 250 W (Fig 6(b)), severe ablation of carbon fibers was observed on both sides of the groove along both orthogonal fiber directions, indicating intensified thermal degradation and enhanced material removal. This trend became more pronounced at 500 W (Fig 6(c)), where extensive fiber ablation and more severe structural damage were evident.

**Fig 6:**
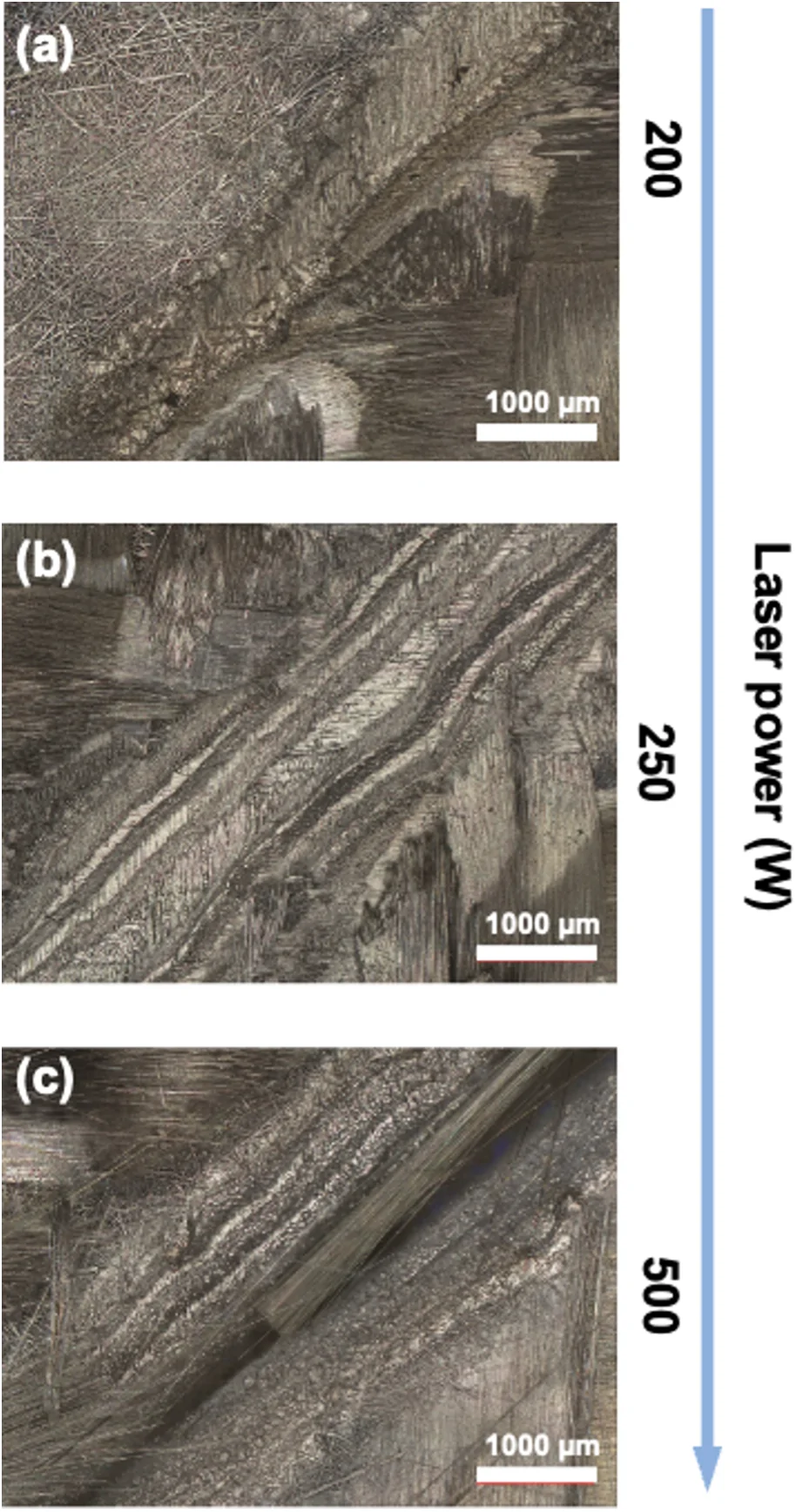
Surface morphologies of laser-cut CFRP specimens at a 45° scanning angle as a function of laser power. (a) 200 W; (b) 250 W; (c) 500 W.

Overall, increasing laser power significantly intensified thermal erosion of the CFRP laminate and promoted heat-affected zone expansion. Similar to conventional laser processing, hybrid laser‑gas‑waterjet machining of CFRP also leads to matrix decomposition and fiber exposure due to thermal conduction, giving rise to the so‑called “matrix recession” defect [31]. Moreover, gas pressure, as a critical process parameter, plays a significant role in regulating the degree of fiber exposure and the surrounding morphological characteristics.

Fig 7 presents the three-dimensional surface topography of laser-cut CFRP specimens processed at a 45° scanning angle. Unlike the 90° configuration, in which the laser traverses strictly perpendicular to one fiber family, the oblique scanning path at 45° simultaneously intersects both orthogonal fiber bundle families of the woven architecture at identical angles, producing a characteristically asymmetric damage distribution along the groove flanks. This geometric asymmetry is clearly reflected in the height maps: the deepest ablation zones (blue and green) are elongated diagonally rather than symmetrically bounded, while the elevated ridges (yellow and red) exhibit alternating periodicity corresponding to the interlacing pitch of the woven preform. Quantitatively, the peak-to-valley height increased from 615.661 μm at 200 W (Specimen 5−2) to 1486.528 μm at 250 W (Specimen 6−2), and further to 2698.104 μm at 500 W (Specimen 7−2), confirming the progressive intensification of material removal with increasing energy input. Notably, the rate of height increase between 250 W and 500 W is substantially steeper than that observed between 200 W and 250 W, suggesting a threshold-like transition in the ablation regime as incident power increases. Taken together, these observations indicate that 45° scanning introduces a geometrically coupled damage pathway absent in purely transverse or longitudinal configurations, in which neither fiber family is afforded effective thermal protection through preferential heat channeling, resulting in broader interfacial disruption for a given energy input compared with the fiber-aligned scanning case. Research has shown that the laser scanning direction—defined as the angle relative to the fiber axial direction in unidirectional CFRP—significantly influences the surface treatment quality and subsequent bonding performance [32]. When the scanning direction is parallel to the fiber orientation (0°), heat cannot be effectively conducted along the fiber axis, leading to intensified local heat accumulation, which in turn causes thermal damage such as fiber fracture and warpage.

**Fig 7:**
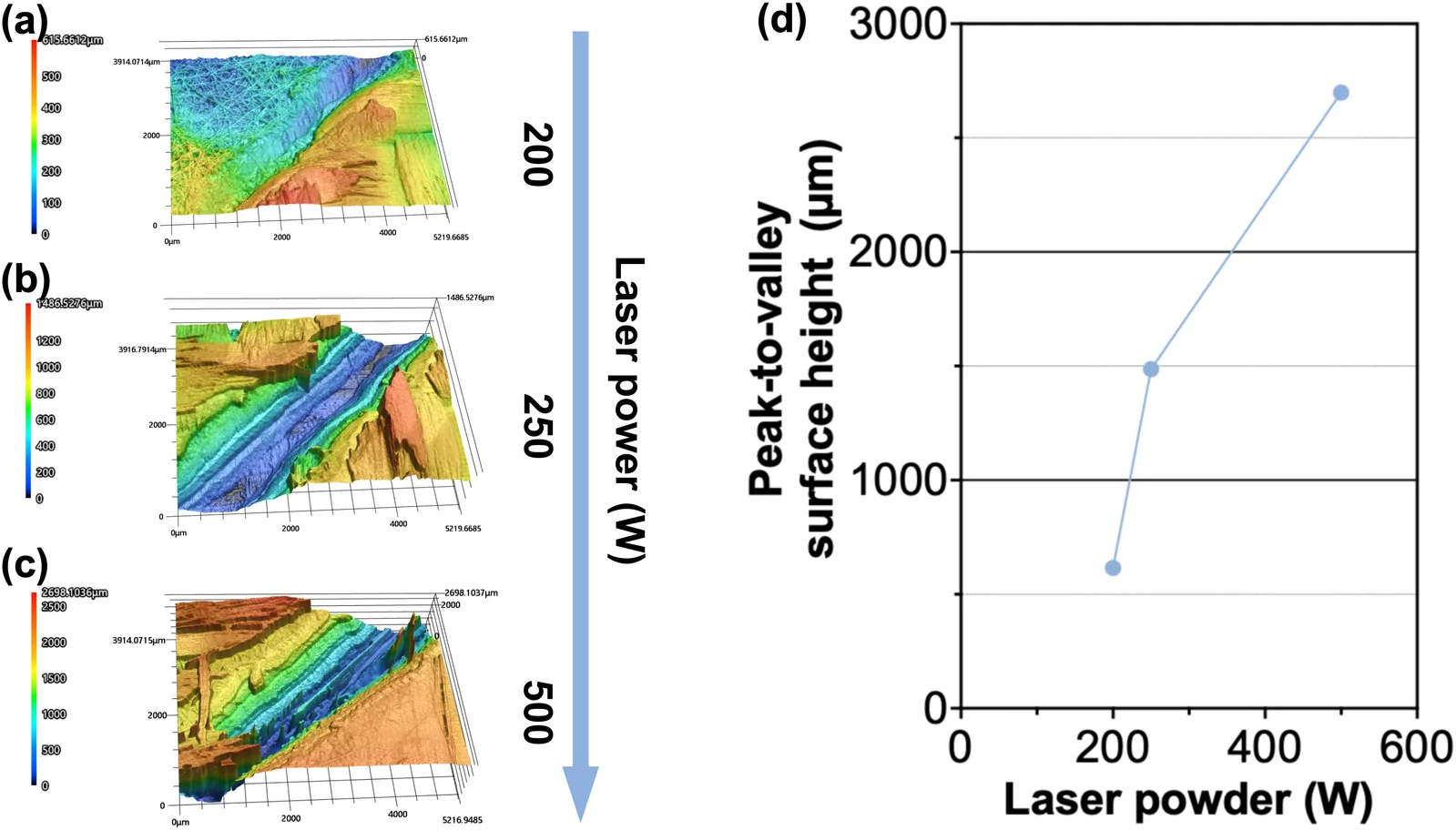
Three-dimensional surface topography of laser-cut CFRP specimens at a 45° scanning angle as a function of laser power. (a) 200 W; (b) 250 W; (c) 500 W; (d) Peak-to-valley surface height trends.

### 3.2 Mechanical response and residual tensile properties

Fig 8 presents the tensile force–displacement curves obtained from mechanical testing. Both intact (unperforated) and laser-drilled specimens exhibit two distinct stages: an initial linear stage with a positive slope corresponding to elastic deformation, followed by a nonlinear stage with a negative slope representing progressive load reduction until final fracture. For the laser-drilled specimens, the average failure load is closely governed by the laser processing parameters. At a fixed laser power (e.g., 1000 W), the failure load decreases monotonically as irradiation time increases from 0.50 s to 1.00 s. Similarly, at a fixed irradiation duration, the failure load decreases with increasing laser power from 250 W to 1000 W. This trend is attributed to more extensive thermal damage to the carbon fibers and a larger heat-affected zone under higher power and longer irradiation, both of which reduce the load-bearing capacity of the specimens. Furthermore, all laser-drilled specimens exhibit lower tensile strength than their unperforated counterparts, owing to stress concentration at the hole edges. Fracture observations reveal that failure in perforated specimens initiates and propagates along cracks at the hole boundaries, whereas in unperforated specimens, fracture originates from the central region of the CFRP plate.

**Fig 8:**
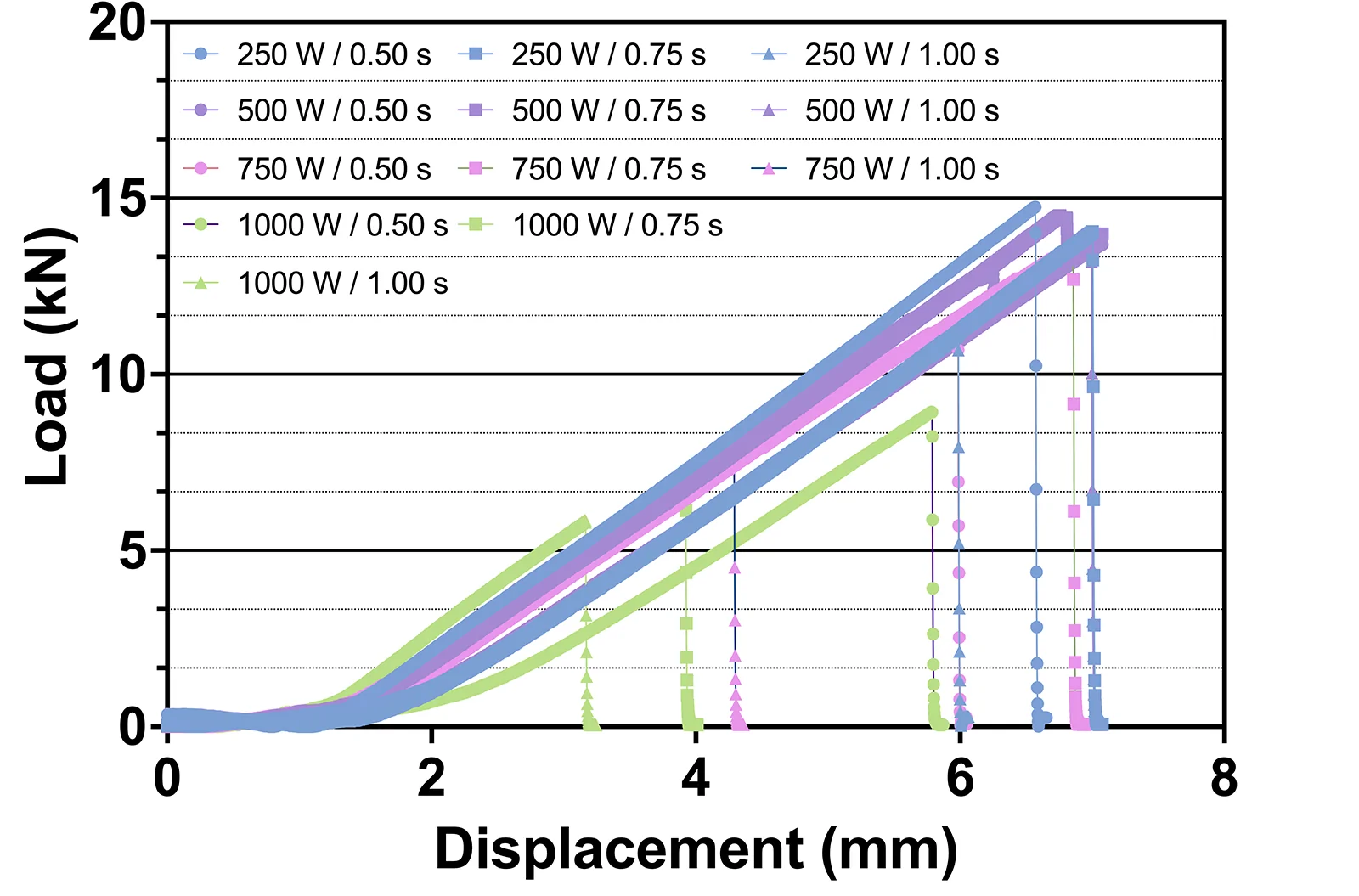
Typical load–displacement curves from tensile testing of laser-drilled CFRP specimens as a function of laser power and ablation duration.

Fig 9 illustrates the influence of laser processing parameters—specifically power and irradiation time—on the tensile strength of carbon fiber-reinforced composites. Both factors exert a significant and synergistic effect on mechanical performance. At low laser power (250–500 W) with brief exposure (0.50 s), tensile strength remains high (~700–750 MPa), indicating that the thermal input is insufficient to induce substantial matrix degradation or fiber–matrix interfacial damage. As laser power increases or irradiation time is prolonged, however, a pronounced strength reduction is observed, reaching approximately 300 MPa at 1000 W and 1.00 s. This dramatic decline is attributable to localized thermal decomposition of the polymer matrix, partial resin carbonization, and weakened fiber–matrix adhesion, which collectively impair load transfer efficiency. In the intermediate regime (500–750 W, 0.75–1.00 s), a gradual reduction of 10–30% is observed, reflecting the sensitivity of these composites to cumulative thermal effects. Excessive laser energy input not only promotes resin degradation but may also introduce microstructural defects—including fiber pull-out, void formation, and delamination—all of which directly compromise tensile performance. These findings underscore the importance of careful parameter optimization: low power combined with short irradiation time enables targeted surface modification without significantly undermining structural integrity, providing practical guidance for laser-based processing strategies aimed at tailoring composite properties while preserving mechanical robustness. Similar studies have demonstrated that the mechanical property degradation of CFRP under laser irradiation follows distinct patterns [33]: with increasing irradiation time, the tensile strength decreases continuously and significantly, whereas the decline in modulus tends to level off; with increasing power density, both strength and modulus deteriorate sharply and simultaneously. The underlying mechanism of this degradation is that laser ablation causes the resin to lose its binding and load-transfer functions, leaving the tensile load to be borne solely by the carbon fibers. At higher power densities, the fiber bundle structure itself undergoes degradation and even linear ablation. These degradation trends and the associated mechanisms are in good agreement with the experimental results obtained in the present study.

**Fig 9:**
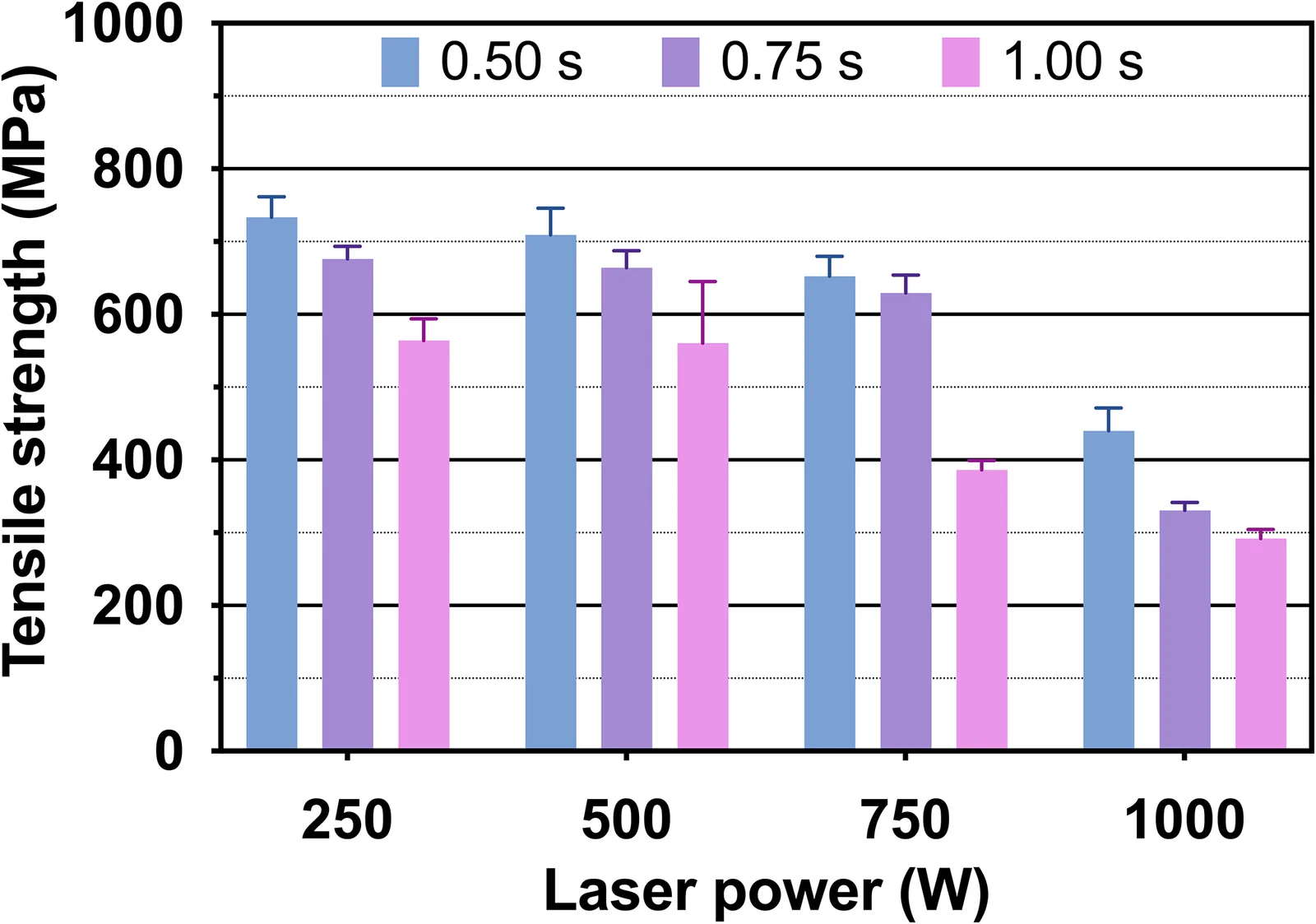
Tensile strength of laser-drilled CFRP specimens as a function of laser power and ablation duration.

Fig 10 presents the tensile force–displacement curves of laser-cut CFRP specimens. All specimens exhibit two characteristic stages: an initial linear segment with a positive slope corresponding to elastic deformation, followed by a nonlinear segment with a negative slope representing progressive load reduction through the plastic damage region until final fracture. The average failure load is closely governed by the cutting parameters. At a fixed laser power (500 W), the failure load decreases as the scanning angle increases from 0° to 90°. Similarly, at a fixed scanning angle, the failure load decreases monotonically as laser power increases from 200 W to 500 W. These trends are attributed to two concurrent mechanisms: higher laser power induces more severe thermal damage to the carbon fibers and enlarges the heat-affected zone, while larger scanning angles—particularly those approaching 90°—orient the cut path increasingly perpendicular to the primary load-bearing fiber direction, severing more load-carrying fibers and substantially reducing the specimen's load-bearing capacity. Furthermore, compared with unperforated and laser-drilled specimens, the laser-cut specimens exhibit lower tensile strength overall, owing to the notch effects and stress concentration introduced by the cutting region.

**Fig 10:**
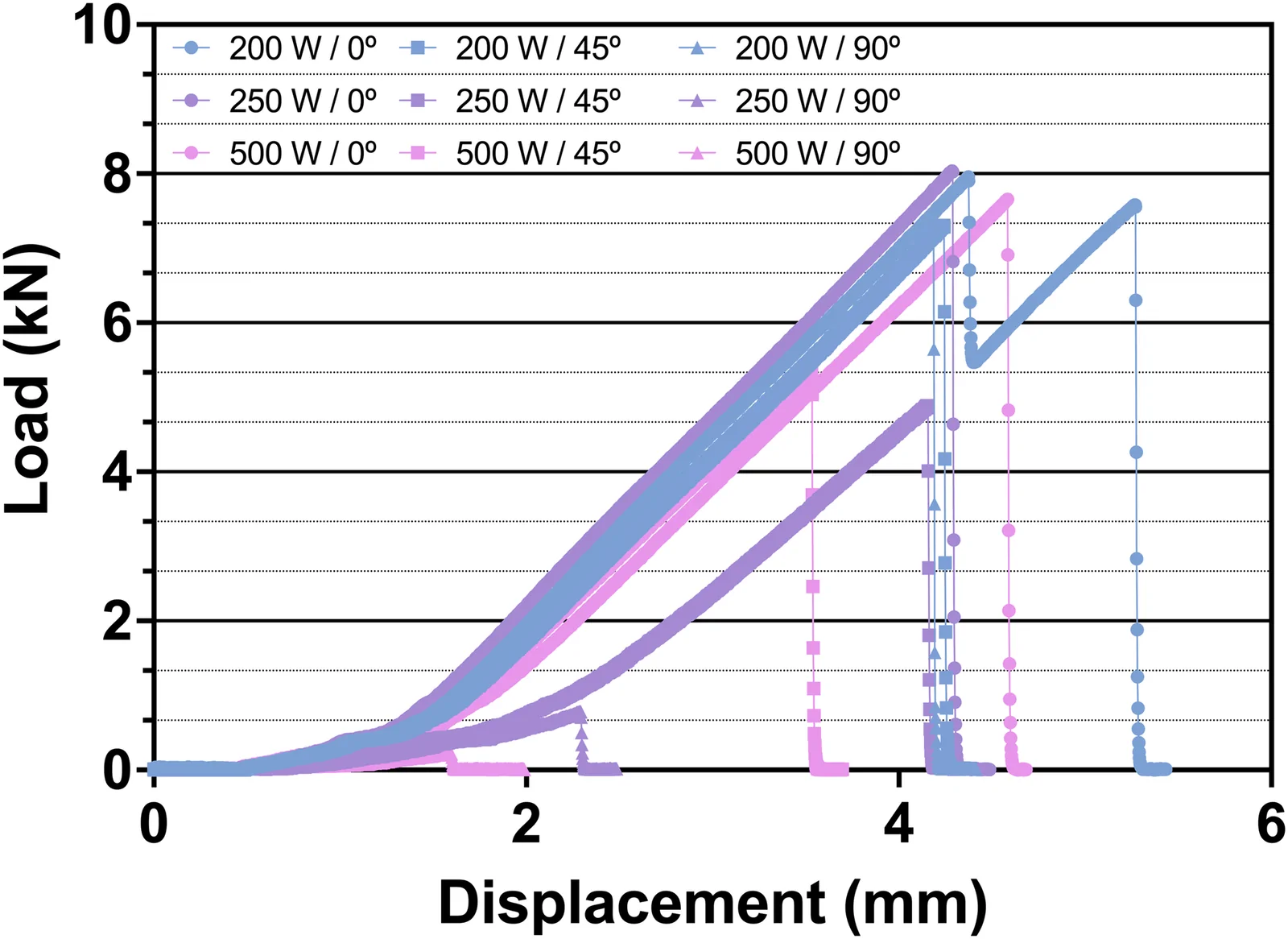
Typical load–displacement curves from tensile testing of laser-cut CFRP specimens as a function of laser power and scanning angle.

Fig 11 presents the tensile strength of carbon fiber-reinforced composites subjected to laser scanning along different orientations (0°, 45°, and 90°) over a distance of 10 mm, as a function of laser power. The results reveal a pronounced dependence of mechanical performance on both laser power and scanning direction. Tensile strength remains relatively high when scanning proceeds along the fiber direction (0°), even at moderate power levels, whereas deviations from the fiber axis—particularly at 90°—lead to substantial strength reductions, with the most severe degradation occurring at high power. This behavior is attributed to the anisotropic thermal response of the composite. Scanning along the fiber axis promotes heat dissipation through the high-conductivity carbon fibers, thereby minimizing local resin decomposition and fiber–matrix interfacial damage. Scanning perpendicular to the fibers, by contrast, concentrates thermal energy within the resin-rich inter-fiber regions, promoting polymer carbonization, interfacial debonding, microcrack formation, and void nucleation—all of which impair load transfer efficiency. The synergistic influence of laser power and scanning orientation highlights the necessity of carefully controlling processing parameters to achieve targeted surface modifications while preserving the structural integrity of the composite.

**Fig 11:**
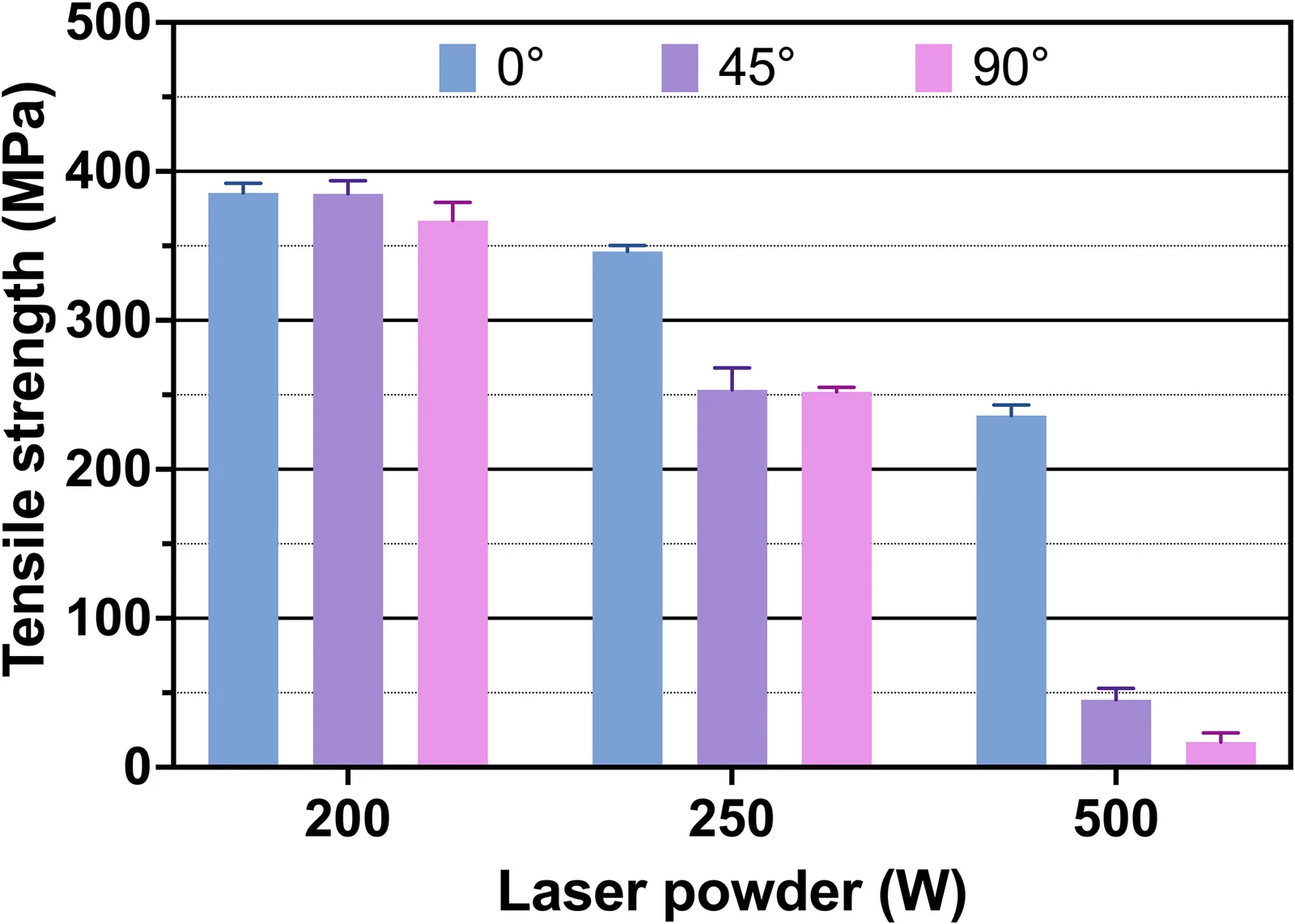
Tensile strength of laser-cut CFRP specimens as a function of laser power and scanning angle.

### 3.3 Damage modeling and predictive framework

To quantitatively characterize laser-induced degradation of CFRP laminates under varying processing conditions, a CDM-based empirical model was developed, as expressed in Equations (2) and (3). Based on the foregoing analysis, the damage variable (D) exhibits pronounced sensitivity to both laser processing parameters and the thermophysical properties of the constituent materials, with its evolution governed by the coupled interaction of multiple contributing factors. Accordingly, the model integrates four key laser processing parameters—power (P), irradiation time (t), scanning angle (θ), and scanning distance (l)—into a unified power-law framework, enabling consistent description of both laser drilling and laser cutting regimes. Nonlinear least-squares regression against all 21 experimental conditions yielded fitted parameters A = 0.0266187, m = 0.464431, n = 0.645373, α = 0.0745099, and β = 0.22664, with a coefficient of determination R^2^ = 0.9151, indicating acceptable agreement between predicted and experimentally measured damage variables. R^2^ quantifies the proportion of the total variation in the dependent variable that is explained by the independent variables in a regression model. In linear regression, R^2^ typically takes values in the range [0,1]; the closer the value is to 1, the better the goodness of fit, although one should also be cautious of overfitting.

The physical significance of each parameter is consistent with the underlying laser–material interaction mechanisms. The power exponent m = 0.464431 reflects a moderate yet persistent sensitivity of damage evolution to laser power: as P increases from 200 W to 1000 W, the elevated energy density intensifies localized photothermal heating, accelerating epoxy matrix decomposition and promoting progressive fiber breakage within the heat-affected zone, behavior consistent with the monotonic increase in D observed across all power levels in Tables 4 and 5. The time exponent n = 0.645373 indicates sub-linear damage accumulation with increasing irradiation duration, attributable to the competition between continuous heat input and thermal diffusion, as well as the progressive depletion of the most thermally susceptible matrix constituents during early irradiation, which yields diminishing incremental damage at longer exposure times.

**Table 4:** Damage variable of CFRP under different laser drilling conditions.

Damage variable		Laser power (W)			
		250	500	750	1000
Scanning time (s)	0.50	0.231310273	0.256813417	0.316215933	0.538784067
	0.75	0.2910587	0.303983229	0.340324948	0.653385744
	1.00	0.408805031	0.412651992	0.595041929	0.693920335

**Table 5:** Damage variable of CFRP under different laser cutting conditions.

Damage variable		Laser power (W)		
		200	250	500
Scanning angle (º)	0	0.596090147	0.637316562	0.752620545
	45	0.596436059	0.734454927	0.952484277
	90	0.615303983	0.735849057	0.982180294

The geometric contribution is represented by the coupled term (1 + l)^β(1 + lsin(θ))^α, where α = 0.0745099 governs the anisotropic correction associated with the combined effects of scanning distance and fiber orientation, while β = 0.22664 characterizes the influence of scanning distance on HAZ development. When θ = 0°, sinθ = 0 and the geometric term reduces to unity, indicating negligible directional contribution to damage—consistent with the weak anisotropic response observed under fiber-parallel irradiation. As θ approaches 90°, sinθ approaches unity, reflecting enhanced fiber–matrix interfacial disruption caused by transverse cutting across fiber bundles and resulting in greater damage accumulation at otherwise identical processing conditions. The small magnitude of α further suggests that, although geometric effects are statistically significant, their contribution remains secondary to the dominant thermal effects governed by laser power and exposure time—physically reasonable given that thermal degradation is the primary failure mechanism in laser–CFRP interactions. A study on the laser drilling of kenaf/HDPE composites similarly demonstrated that laser power and cutting speed significantly affect the formation of the heat-affected zone [34]. Increasing the laser power enhances energy absorption, thereby intensifying material degradation and enlarging the heat-affected zone, whereas reducing the cutting speed prolongs the laser–material interaction and consequently promotes heat accumulation.

Taken together, the physically interpretable magnitudes and signs of all fitted exponents, combined with the acceptable R^2^ value, confirm that the proposed model provides an experimentally validated quantitative framework for predicting laser-induced damage in CFRP laminates. The unified formulation successfully bridges drilling and cutting regimes, offering a consistent tool for structural integrity assessment across diverse laser processing conditions.


$$\begin{array}{c} \begin{array}{c} D=\text{1}-\sigma /\sigma_{\text{0}} \end{array} \end{array}$$ (2)

$$\begin{array}{c} \begin{array}{c} D(P,t,\theta ,l)=AP^{m}t^{n}{\left(\text{1}+\text{l}\cdot \text{sin}\theta \right)}^{\alpha }(\text{1}+\text{l}{)}^{\beta } \end{array} \end{array}$$ (3)

Fig 12 compares the predicted and measured damage variables under various laser drilling and cutting conditions using a single set of model parameters. Across all 21 observations, the model achieves a Pearson correlation coefficient of 0.9566, a mean absolute error (MAE) of 0.04995, and an R^2^ of 0.9151, indicating good overall agreement. The fitting performance nevertheless differs between the two processes. For laser drilling, the predictions exhibit greater scatter around the 1:1 line, with r = 0.8758, MAE = 0.06260, and R^2^ = 0.7613 The largest absolute deviation occurs at 750 W and a scanning time of 0.75 s, where the model predicts a damage variable of 0.47844 compared with the measured value of 0.34032, corresponding to an overestimation of 0.13811 (40.58%). Conversely, the largest underestimation occurs at 1000 W and 0.50 s, with predicted and measured values of 0.42092 and 0.53878, respectively. The laser cutting data show closer agreement with the 1:1 line, yielding r = 0.9661, MAE = 0.03309, and R^2^ = 0.9193. The largest absolute deviation within this group occurs at 500 W and a scanning angle of 0°, where the predicted value of 0.82167 exceeds the measured value of 0.75262 by 0.06905 (9.17%). These results indicate that the shared-parameter model represents the cutting data more accurately than the drilling data, while retaining appreciable local discrepancies under certain drilling conditions. The reported metrics describe agreement with the calibration data rather than independent predictive validation.

**Fig 12:**
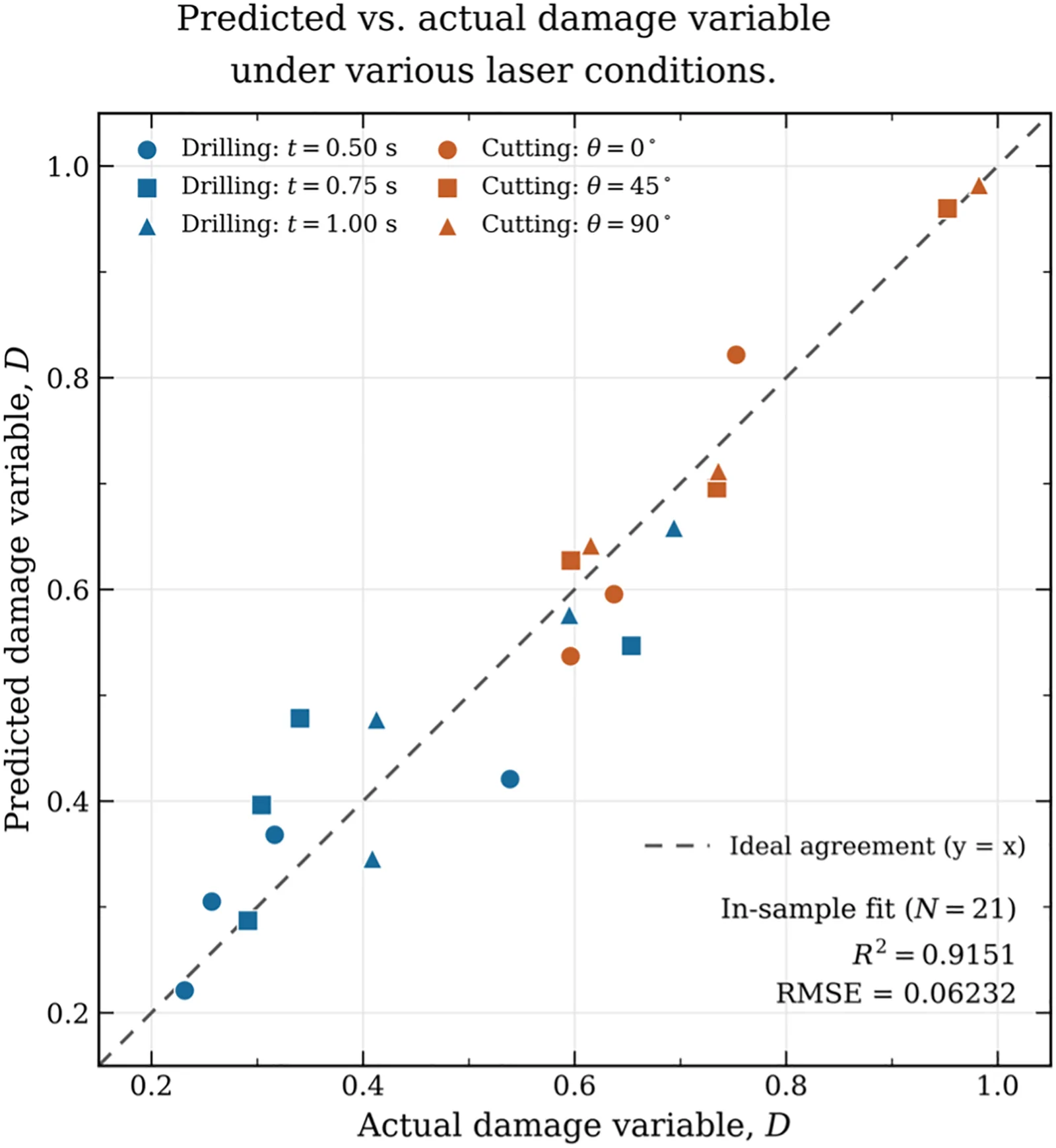
Predicted vs. actual damage variable under various laser conditions.

To assess the predictive capability of the proposed model, additional CFRP experiments were conducted under three processing conditions excluded from the calibration dataset: one laser-drilling condition (600 W, 1 s) and two laser-cutting conditions (225 W at 45° and 400 W at 90°). Three replicate measurements were obtained for each condition, and the tensile strengths are summarized in Table 6. The model reproduces the decrease in tensile strength across these conditions, with a mean absolute error of 15.03 MPa relative to the experimental averages. For laser drilling at 600 W, the predicted strength of 458.55 MPa agrees closely with the measured average of 450.33 MPa, corresponding to an overestimation of 1.83%. For laser cutting at 225 W and 45°, the model predicts 321.93 MPa, exceeding the measured average of 303.67 MPa by 6.01%. The largest relative deviation occurs at 400 W and 90°, where the predicted strength of 109.05 MPa underestimates the measured average of 127.67 MPa by 14.58%. This independent comparison supports the model’s predictive capability within the tested conditions, although its accuracy is lower for the condition yielding the lowest tensile strength.

**Table 6:** Comparison of experimental and predicted results for an independent dataset.

Power (W)	Angle (º)	Time (s)	Test1 (MPa)	Test2 (MPa)	Test3(MPa)	Average (MPa)	Prediction (MPa)
600	0	1	443	446	460	450.33	458.55
225	45	1	292	304	315	303.67	321.93
400	90	1	122	127	134	127.67	109.05

Existing prediction approaches range from analytical heat-conduction models to three-dimensional transient thermal simulations and coupled process–structure damage models. Takahashi et al. developed a heat-conduction model to describe thermal transport within the carbon fibers and polymer matrix [35], whereas Keuntje et al. employed a three-dimensional finite-element model to predict temperature evolution and material removal during CFRP laser cutting [36]. Schmidt et al. further coupled thermal simulation with anisotropic damage modelling to evaluate the effects of the HAZ on tensile failure [37]. In addition, Tao et al. used an energy-based continuum damage mechanics model to predict the residual tensile strength of CFRP laminates containing drilling-induced delamination [38]. These approaches provide detailed physical descriptions but generally require extensive material parameters and considerable computational effort. In contrast, the present empirical CDM-based model provides a computationally efficient means of predicting the overall damage induced by both laser drilling and laser cutting directly from the processing parameters.

From an industrial perspective, the present results provide practical guidance for balancing processing efficiency and structural integrity during laser machining of CFRP components. The laser energy input should be sufficiently high to ensure complete and stable material removal but should not exceed the level at which excessive matrix decomposition, fiber ablation, and heat-affected-zone expansion occur. Based on the present results and under the current equipment and experimental conditions, a laser power of 1000 W and an irradiation time of 0.75 s are recommended for laser drilling, as this combination enables complete hole formation while limiting the extent of the heat-affected zone. For laser cutting, 500 W provided the most favorable processing performance within the investigated range and is therefore considered the optimum setting. However, the influence of the scanning-path angle on the size and distribution of the HAZ should also be carefully considered during parameter selection.

Given the current scope of the study and the limited sample size, we primarily focused on elucidating the relationship between damage characteristics and mechanical performance. Accordingly, a CDM approach was adopted to predict the mechanical response. As larger datasets and more comprehensive features become available, machine-learning methods will be introduced to enable more in-depth analyses and uncover complex underlying relationships [39]. Future research will also extend the proposed framework to more complex laser-scanning paths and the mechanical response of large-scale CFRP components.

## 4 Conclusion

In this study, the microstructural evolution, mechanical degradation, and damage modeling of CFRP laminates subjected to laser drilling and laser cutting were systematically investigated. The following conclusions can be drawn:

(1) Laser power and irradiation duration synergistically governed the thermal damage of laser-drilled CFRP. Increasing laser power from 250 W to 1000 W enlarged the ablation crater diameter by approximately 200% (1007 μm to 3021 μm), while extending irradiation time from 0.5 s to 1.0 s expanded the crater by 37%. The surface height difference increased from 646 μm to 3392 μm under extreme conditions, with through-thickness perforation observed at high power and prolonged exposure, confirming that thermal damage propagates beyond the direct irradiation zone via carbon fiber thermal conductivity.
(2) For laser-cut specimens, increasing power from 200 W to 500 W transformed the fiber fracture morphology from arc-shaped to flat and uniform, with full laminate penetration achieved at 500 W. The maximum surface height difference increased from 588 μm to 4259 μm at 90° scanning. Scanning perpendicular to the fiber direction induced broader heat-affected zones and more severe interfacial disruption than fiber-parallel scanning, reflecting the anisotropic thermal conductivity of the woven CFRP architecture.
(3) Residual tensile strength decreased monotonically with increasing laser energy input across both processing modes. For drilled specimens, strength declined from approximately 735 MPa (250 W, 0.5 s) to 293 MPa (1000 W, 1.0 s), a reduction exceeding 60% relative to the pristine laminate strength of 954 MPa. For cut specimens, strength at 500 W decreased from 466 MPa at 0° to 233 MPa at 90°, attributable to the combined effect of intensified thermal fiber damage at higher power and the progressive severing of load-bearing fibers as scanning angle approached perpendicularity to the fiber axis. Laser-cut specimens exhibited systematically lower residual strength than drilled counterparts under comparable power conditions, consistent with the additional stress concentration introduced by the linear cutting kerf. These trends are reflected in the damage variable D, which ranged from 0.231 to 0.694 for drilled specimens and from 0.596 to 0.982 for cut specimens.
(4) A CDM-based empirical model was established to unify damage prediction across drilling and cutting regimes. Fitted parameters (A = 0.0266187, m = 0.464431, n = 0.645373, α = 0.0745099, and β = 0.22664) achieved R^2^ = 0.9151 over 21 test conditions. The sub-linear exponents physically reflect diminishing marginal damage accumulation, while the geometric term captures anisotropic angular dependence, providing a validated predictive tool for structural integrity assessment under diverse laser processing conditions.

The following abbreviations are used in this manuscript as shown in Table 7

**Table 7:** Abbreviations of terms.

Number	Abbreviated terms	Full written terms
1	3D	Three-dimensional
2	CDM	Continuum damage mechanics
3	CFRP	Carbon fiber-reinforced polymer
4	CW	Continuous wave
5	HAZ	Heat-affected zone
6	MAE	Mean absolute error
7	MLT	Matzenmiller–Lubliner–Taylor model
8	RH	Relative humidity
9	RMSE	Root mean square error
10	SEM	Scanning electron microscopy
11	UV	Ultraviolet
